# Tracking an Elusive Killer: State of the Art of Molecular-Genetic Knowledge and Laboratory Role in Diagnosis and Risk Stratification of Thoracic Aortic Aneurysm and Dissection

**DOI:** 10.3390/diagnostics12081785

**Published:** 2022-07-22

**Authors:** Rosina De Cario, Marco Giannini, Giulia Cassioli, Ada Kura, Anna Maria Gori, Rossella Marcucci, Stefano Nistri, Guglielmina Pepe, Betti Giusti, Elena Sticchi

**Affiliations:** 1 Department of Experimental and Clinical Medicine, University of Florence—Atherothrombotic Diseases Unit, Careggi University Hospital, 50134 Florence, Italy; rosina.decario@unifi.it (R.D.C.); m.giannini16@student.unisi.it (M.G.); g.cassioli2@student.unisi.it (G.C.); ada.kura@unifi.it (A.K.); annamaria.gori@unifi.it (A.M.G.); rossella.marcucci@unifi.it (R.M.); guglielmina.pepe@unifi.it (G.P.); elena.sticchi@unifi.it (E.S.); 2 Department of Cardiology, CMSR, 36077 Altavilla Vicentina, Italy; stefanonistri41@gmail.com; 3 Reference Regional (Tuscany) Center for Marfan Syndrome and Related Disorders, Largo Brambilla 3, 50134 Florence, Italy

**Keywords:** thoracic aortic aneurysm and dissection, syndromic aortopathies, differential diagnosis, genetics, biomarkers, genetic diagnosis, review

## Abstract

The main challenge in diagnosing and managing thoracic aortic aneurysm and dissection (TAA/D) is represented by the early detection of a disease that is both deadly and “elusive”, as it generally grows asymptomatically prior to rupture, leading to death in the majority of cases. Gender differences exist in aortic dissection in terms of incidence and treatment options. Efforts have been made to identify biomarkers that may help in early diagnosis and in detecting those patients at a higher risk of developing life-threatening complications. As soon as the hereditability of the TAA/D was demonstrated, several genetic factors were found to be associated with both the syndromic and non-syndromic forms of the disease, and they currently play a role in patient diagnosis/prognosis and management-guidance purposes. Likewise, circulating biomarker could represent a valuable resource in assisting the diagnosis, and several studies have attempted to identify specific molecules that may help with risk stratification outside the emergency department. Even if promising, those data lack specificity/sensitivity, and, in most cases, they need more testing before entering the “clinical arena”. This review summarizes the state of the art of the laboratory in TAA/D diagnostics, with particular reference to the current and future role of molecular-genetic testing.

## 1. Introduction

Despite the considerable advancements pursued over the past decades in pathophysiology knowledge and diagnostic imaging techniques for thoracic aortic aneurysm (TAA), plus the more consistent surveillance programs after diagnosis, it remains a life-threatening and still “subtle” disease for which the true epidemiology is hard to be determined. Ninety-five percent of TAA patients do not show any symptoms unless an acute aortic event occurs [1]. Thus, the estimated prevalence remains at 6–10 cases/100,000 patients/year, with ascending TAAs being more common compared to descending TAAs (60% vs. 35%) [2]. The most feared consequences of TAA are represented by aortic dissections/ruptures, the most common of the catastrophic events affecting the aorta [3]. In this respect too, the true incidence of aortic dissection is not easy to assess, as hospital-based reports clearly do not account for pre-admission deaths, which are known to involve a substantial proportion of TAA individuals [4]. Gender differences in aortic dissection were observed in a recent Swedish population-based study, estimating a decreasing incidence in men during 15 years. Differently, in women, the incidence did not show a significant variation. Moreover, fewer women were treated with thoracic endovascular aortic repair (TEVAR), and they showed a higher postoperative mortality when compared to men [5].

TAA has traditionally been divided based on the presence of extra-aortic clinical manifestations—syndromic TAA—or their absence—non-syndromic TAA. Syndromic TAA patients present with systemic features reflecting the presence of diseases involving connective tissue disorders such as Marfan syndrome (MFS), Loeys-Dietz syndrome (LDS), vascular Ehlers-Danlos syndrome (vEDS), and arterial tortuosity syndrome [6]. Bicuspid aortic valve (BAV) represents another condition often found in association with TAA, which is currently considered an independent risk factor for its development, with the aorta being dilated in some cases, even in the presence of a normally functioning BAV and/or regardless of physical activity, as has been recently assessed in pediatric patients [7,8]. Non-syndromic TAAs account for 95% of all TAA cases [6] and are further classified as sporadic or familial, in the case of at least one of the first-degree family members being affected [9]. The different presentations of TAA share similarities with regards to the molecular pathophysiology underlying dilatation development (impaired extracellular matrix (ECM)), collagen homeostasis, alteration of the TGF-β signaling pathways, disruption of smooth muscle and cytoskeletal apparatus, and, even if with different penetrance, a significant heritability [10].

According to the ESC (European Society of Cardiology) guidelines, TAA presentation has an impact on management, measures of intervention, and therapeutic strategies, as aortic growth speed and progression vary between syndromic and non-syndromic cases and also between sporadic and familial ones [11]. Thus, early identification of asymptomatic patients is crucial to allow timely monitoring and management strategies that could potentially prevent the progression of TAA and TAD [6]. This is especially important in non-syndromic cases in which the lack of distinct clinical manifestations (involving musculoskeletal or ocular signs) may delay detection. From the laboratory point of view, given that syndromic/non-syndromic TAA are most often inherited in an autosomal dominant pattern, molecular-genetic analysis represents a most valuable tool allowing early identification of affected individual as well as those subjects at higher risk of developing adverse outcomes in terms of aneurysm-growth speed and dissection [12]. This is particularly relevant for TAAs involving the ascending aorta, whose etiology is predominantly linked to a crucial genetic component. In addition, easily detectable and specific circulating biomarkers might further improve diagnosis and overall management of the disease. This review will address the state of the art of TAA’s pathophysiological drivers and related current laboratory tests, as well as genetic approaches and their major implications (most particularly regarding novel technologies/strategies) in supporting the detection and management of a condition with features (slow, gradual, and painless aneurysm formation, usually “accidentally” diagnosed via imaging study carried out for another purpose), that have made it earn, over the years, the sinister reputation of a “silent killer” [13].

## 2. Drivers of TAA Formation: A Constant Journey through Gene Discovery

The complexity and heterogeneity of TAA characteristics, syndromic presentation, and/or progression is the consequence of multiple but unique cellular and molecular-genetic mechanisms underlying its development, which often result in similar clinical presentation [14]. As familial-aggregation studies have suggested, more than 20% of patients have at least one first-degree family member with an arterial aneurysm, basically defining an increased risk for relatives of the affected individuals [15]. The first clue about the heritability of the trait is derived from case-control studies comparing the prevalence of thoracic aortic aneurysms, thoracic aortic dissections, and sudden death in first-degree relatives of patients referred for thoracic aortic surgery [16], identifying a higher risk for developing those diseases in the proband first-degree relatives with respect to the control groups (with relative risks of 1.8, 10.9, and 1.8 in proband fathers, brothers, and sisters, respectively). More evidence on genetic factors contributing to TAA development was provided by an analysis of a database comprising 598 patients evaluated for TAA in the United States [17], which showed a faster growth rate of aortic aneurysm in patients with familial cases with respect to the sporadic ones, with a younger age of presentation. In addition, pedigrees also showed different patterns of inheritance (autosomal dominant, X-linked, autosomal recessive). The role of genetic factors in causing TAA was further confirmed by more recent studies as well, analyzing different type of aneurysms [18]. It was mainly through genetic and animal models’ studies that the combination of disrupted/altered cellular processes driving the TAA formation were elucidated as well as the specific associated genes (Table 1). In this regard, it has to be noticed, which some causative genes exert an overlapping effect on both syndromic and non-syndromic TAAs, even if these two conditions have traditionally been considered as distinct entities (e.g., ACTA2, SMAD3) (Table 1) [6].

### 2.1. Extracellular Matrix Components

**Figure 1:**
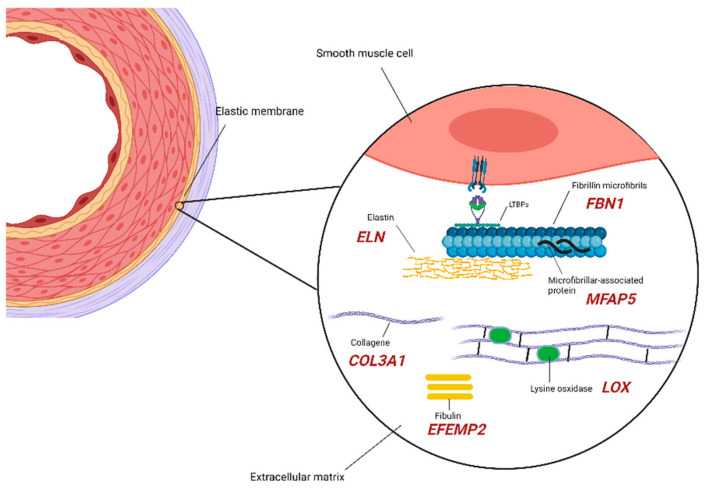
Schematization of the ECM main components. Genes codifying each component are reported in red (created with BioRender.com (accessed on 13 May 2022)).

Among the genes causing and/or influencing TAA development, those codifying the ECM components are always mentioned first due to the amount of data that was collected over the years through animal studies, providing evidence of their impact on maintaining the structural integrity of the aortic wall. Those components are in close relationship and represent key factors, upstream/downstream, and intermediate elements of cellular pathways with impairment that has been demonstrated to have consequences in aneurysm development/predisposition in different ways, namely depletion of the elastic lamina in the aortic wall, lengthening of the ascending aorta, impaired assembly of collagen and elastic fibers, and altered TGF-β signaling (Figure 1).

The *FBN1* gene (15q21.1) encodes the key component of extracellular microfibrils fibrillin-1, with a major role in elastin (encoded by *ELN* gene, 7q11.23) assembly and support by promoting adhesion to vascular smooth muscle cells (VSMCs) through interaction with lysyl oxidase (encoded by *LOX* gene, 5q23.1) and fibronectin [45]. Robust data are available on TAA-causing variants, which were found to be responsible of protein synthesis/secretion’s impairment or incorporation of mutant fibrillin in the microfibrillar architecture [46]. *FBN1* represents the causative gene of Marfan syndrome [47] in syndromic TAA (Table 1), but mutations involving this gene were found in sporadic, non-syndromic TAA as well [25]; besides, a large Whole Exome Sequencing (WES) performed on syndromic and non-syndromic TAAD reported *FBN1* as the most mutated gene of the cohort [26].

As mentioned, fibrillin 1 regulates the assembly of elastin, a protein that, when dysfunctional and/or depleted, has been found in association with TAA, even if additional mechanisms are required to initiate the dilatation development (altered integrins signaling, focal adhesion) [46]. Mutations involving *ELN* gene are causative of cutis laxa, which has an association with aortic dilatation that was found in 30–50% of patients [22]. Regarding the non-syndromic presentation of TAA, a triplication around the elastin gene was found to segregate in a family with supravalvular non-syndromic aortic aneurysm and in which the diagnosis of cutis laxa was excluded [23].

Stabilization and assembly of elastin is also regulated by the enzymatic activity of the lysyl oxidase protein, encoded by the *LOX* gene (5q23.1), catalyzing the lysin residues’ oxidation and those crosslinking reactions required for the stability of the elastin molecules [48]. Inactivating mutations involving the *LOX* gene were found to be causative of TAA development in patients with MFS, familial TAA and dissection (FTAAD), and BAV, through mechanisms that remain to be entirely clarified [28,49].

Fibulin-4 (*EFEMP2 11q13.1*) and type III collagen (encoded by *COL3A1* gene 2q32.2) represent substrates of the enzyme encoded by the *LOX* gene that were shown to promote TAA development as well, when impaired in response to mutations involving the two codifying genes. The matrix glycoprotein fibulin-4 functions as an enhancer of lysyl oxidase activity and as a recruiter of immature elastin molecules: when mutated, the disruption of the elastic fibers and collagens on the aortic wall drives the aneurysm formation, as shown in both human and mouse models [50]. A similar mechanism described for fibulin-4 results from mutations in *COL3A1*, the causative gene of vEDS, with a main function to protect against the catastrophic disruption of the aortic wall that may result from an impaired deposition and maturation of collagen [51], mostly in syndromic forms of TAA [20].

Other ECM key components with dysregulation that has been found to drive TAA formation are the microfibril-associated glycoprotein 2 (*MFAP5* gene, 12p13.3) and biglycan (*BGN*, Xq28). Loss-of-function mutations in the first gene were shown to impair TGF-β and notch-1 signaling, as a consequence of the interaction’s loss with fibrillin-1 [52]. A likely pathogenic variant was identified in a patient with mild TAA restricted to the ascending aorta, BAV, and subtle craniofacial features consistent with a connective-tissue disorder [31]. BGN mutations cause syndromic forms of TAA [19]. Its function is once again related to an alteration of the TGF-β signaling pathways, in which it acts as negative regulator, increasing TGF-β bioavailability [53].

### 2.2. SMCs (Smooth Muscle Cells) Compartment

The aorta is, for the most part, constituted by populations of SMCs, and the maintenance of the contractile properties of their cellular components is highly controlled and regulated. Molecular studies have, in fact, demonstrated that the impairment at different levels of this strictly regulated system predispose individuals to TAA development. Among proteins participating in this cellular machinery, α-smooth muscle aorta encoded by *ACTA2* gene (10q23.31) exerts an important function in maintaining contractility, and its depletion is, in turn, associated with a decrease ability of the monomer to assemble into polymeric filaments, eventually impairing the actin-myosin contractile unit [54]. Decades of study has proven mutations in this gene represent an important cause of familial and nonfamilial non-syndromic TAAD [55,56,57], accounting for 12–21% of TAAD.

Filamin A (*FLNA* gene, Xq28) is an anchoring cytoskeletal protein that, among its diverse functions, acts as a linker between actin filaments [58]. An 18.4% TAA frequency was found in a large systematic analysis of both pediatric and adult patients with periventricular nodular heterotopia carrying loss-of-function *FLNA* mutations [33] and, very recently, in a patient developing TAA with an history of systemic lupus erythematosus [59].

Myosin heavy chain 11 (*MYH11* gene, 16p13.11) interacts with α-actin, controlling its state change and enabling the actin filaments binding. *MYH11* is also traditionally classified as a mostly non-syndromic gene for TAA and was demonstrated to be associated with other manifestations such as aortic stiffness, an early hallmark of the disease [34,60]. However, it has to be noticed that *MYH11* defects account for <1% of all non-syndromic TAAD, with an aorta diameter prior to dissection usually >5 cm [35]. Robust data support the idea of shared defects in *FLNA*, *MYH11*, and *ACTA2* machinery, determining mechanical-strength disruption of the vascular walls and contractility-maintenance failure as significant causes of TAA progression, especially in non-syndromic cases [32].

**Figure 2:**
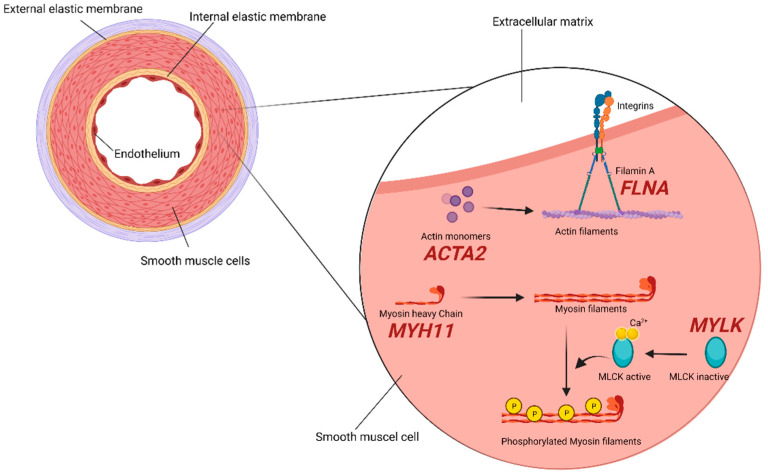
Schematization of the main components of the SMCs compartment. Genes codifying each component are reported in red (created in BioRender.com (accessed on 13 May 2022)).

The *MYLK* gene (3q21.1) encodes the myosin light chain kinase, regulating the actin-myosin interaction and phosphorylating myosin light-chains, and its most destructive mutations were once again mostly found in non-syndromic TAAD [9] (Figure 2).

### 2.3. TGF-β Signaling

**Figure 3:**
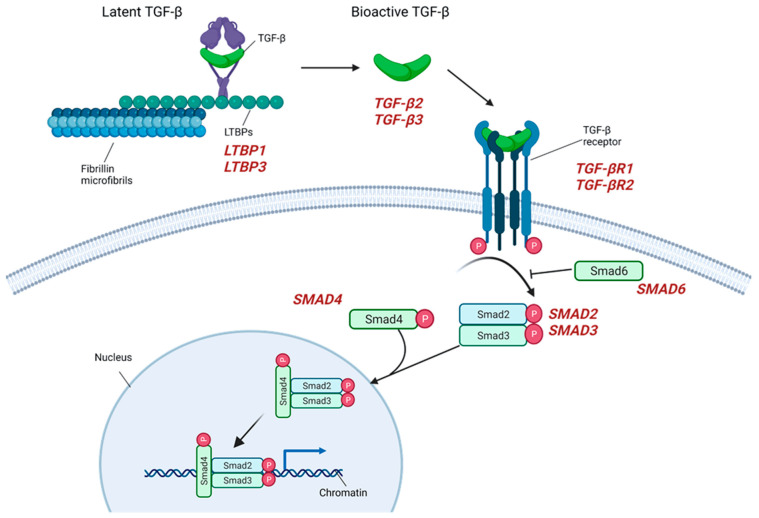
Schematization of the main components of TGF-β signaling. Genes codifying each component are reported in red (created in BioRender.com (accessed on 13 May 2022)).

A mutational repertoire in the genes coding for positive and negative regulators of TGF-β signaling has been reported in patients developing TAA in association with MFS and LDS, with those large volumes of animal studies research representing the first, and one of the major, contributions to our understanding of aneurysmal onset and growth [61]. TGF-β signaling, in fact, plays a critical role in a series of vascular cellular processes such as blood-vessel development and maintenance, positive regulation of contractile proteins’ expression, cell differentiation, proliferation, and homeostasis, and those mechanisms have been demonstrated to be dysregulated in all types of LDSs [61]. Different models have been proposed as the basis of aortic-wall dilatation, one of those suggesting a reduction in TGF-β signaling that would cause an impaired expression of contractile proteins, resembling the effect of mutations in *ACTA2* and *MYH11* genes [62]. Alternatively, LDS-causing mutations increase the VSMCs’ signaling capacity, resulting in a defective responsiveness to the TGF-β of cardiac neural crest-derived VSMCs, which are highly abundant in the proximal thoracic aorta and in excessive activation of TGF-β signaling [63,64]. TGF-β is secreted by many types of cells, including macrophages, as part of a large latent complex that consists of the mature TGF-β cytokine, a dimer of its processed latency associated peptide (LAP), and one of three latent TGF-β binding protein-isoforms (LTBP1, 3, or 4). The latter binds to ECM components such as fibronectin or microfibrils composed of fibrillin-1. Among the LTBP protein family, a deletion involving the *LTBP1* gene (2p22.3) has been found to segregate in a three-generation family presenting with TAA [38], while a variant involving *LTBP3* gene (11q13.1) was suggested to be predisposing to TAA/D development [65]. Upon release, TGF-β binds to its heterodimeric receptor, activating the phosphorylation of the SMAD2 and SMAD3 proteins, which transmit the signal to the nucleus via the association with SMAD4 and, in turn, activate gene transcription [66]. Upon LDS-causing mutations, those affecting elements of the TGF-β signaling pathways involve the receptor heterodimer, made of the two components codified by the *TGFBR1* (9q22.33) and *TGFBR2* (3p24.1) genes; …these mutations result in decreased kinase activity with a reduction in the transduction molecules levels codified by the *SMAD2* (18q21.1) and *SMAD3* (15q22.33) genes [46]. The SMAD2 and SMAD3 proteins belong, in fact, to the receptor-activated (R)-SMAD family, intracellular effectors of the canonical TGF-β signaling pathway, with activated ligands that include the TGF-β2 and TGF-β3 encoded by *TGFB2* (1q41) and *TGFB3* (14q24.3). Mutations involving those genes have been associated with different subtypes of LDS, sharing aneurysm formation as a common clinical manifestation and being characterized by the presence/absence of other systemic features such as aortic or arterial tortuosity, cleft palate, bifid uvula, mitral valve disease, skeletal overgrowth, and so on [39] as well as a different tendency to early aortic dissection. *TGFBR1*, *TGFBR2* and *SMAD3* mutations also account for up to 3%, 5% and 2%, respectively, of non-syndromic FTAAD [32] (Figure 3).

### 2.4. TAA in the Context of Bicuspid Aortic Valve and the Role of Proteases

Since approximately 40% of BAV patients are prone to develop ascending aortic dilatation, this congenital heart defect is currently considered as an independent risk factor for TAA [7,67]. As the two phenotypes frequently occur together, and given the autosomal pattern of inheritance with incomplete penetrance that has been proposed for BAV, hypotheses have been made about the pathophysiological mechanisms driving the valve anomaly, along with its more frequent complication that, among others, involves the potential role of genetic syndromic TAA-associated variants such as *NOTCH1*, *ROBO4*, *SMAD6*, *ELN*, *FBN1*, *ACTA2*, and *LOX* [24,68]. Metalloproteases (MMPs) have been hypothesized to participate in TAA development in the context of BAV, following the observation of a significant increase in MMP-2 levels associated with a reduction in TIMP-1 in BAV/TAA compared with TAA subjects, in the context of a normal tricuspid valve; a greater activity of MMP-2 and MMP-9 in aneurysms was associated with BAV, which could explain the higher prevalence of TAA in these patients [69,70]. Apart from BAV, the combination of altered levels of MMPs, ADAMTS, and TIMPs (the main proteases and inhibitors within the media controlling the ECM environment’s integrity and maintenance) are proven to actively contribute to the medial degeneration triggering TAA [71]. Studies on human and animal models led to the observation of increased expression of MMP-2 and MMP-9 in TAA intima and media [72] and higher levels of ADAMTS-1 and ADAMTS-4 in sporadic, ascending TAA tissues from human patients [73]. Despite the indisputable value of these observation in understanding TAA pathogenesis and their potential implications in diagnosis, it has to be noticed that those markers are not specific for the phenotype, and their levels are altered in several other processes such as AAA (MMP-2, MMP-9), cancer (MMP-9, TIMP-1, TIMP-2), renal disease (TIMP-2), and so on [74] and are currently not included in the recommended work-up for TAA diagnosis and management nor in the genetic screening.

## 3. Mechanisms of TAA Progression: The Dissection Menace

As the pathophysiological mechanisms underlying TAA onset in both its syndromic and non-syndromic presentation have been illustrated, and their specific molecular features continue to be unraveled thanks to the advancements in wide genetic-screening technologies, detection of the disease remains a clinical challenge. This is especially relevant with respect to its most catastrophic complications, rupture and dissection, leading to death in the great majority of patients without timely treatment [4,75]. In fact, once aortic dissection has occurred, mortality is 1–2% for each hour afterwards, resulting in a 48-h mortality of approximately 50% [76]. However, in the case of survival, serious complications may follow such as lethal malperfusion syndrome, aortic regurgitation, cardiac failure, and stroke [77]. Dissection represents a considerable diagnostic challenge for physicians due to the rarity of the condition and the characteristic symptomatology often mimicking other, more common diseases, determining a delay in diagnosis in >30% of cases [4]. Thus, the understanding of the pathophysiology, the key features, and the potential biochemical/molecular markers of TAA progression into aortic dissection has been crucial during the last decades to improve outcomes, for long-term prognosis, and eventually for patients’ risk-stratification purposes.

### 3.1. Pathophysiology and Risk Factors

The instability and the deteriorating integrity of the aortic wall may be due to being predisposed to inherited conditions (such as inherited connective tissue disorders) or can be acquired, as happens with atherosclerotic degeneration due to ageing. Two mechanisms have been proposed to initiate the dissection cascade: (1) in most cases, a tear in the intima exposes the medial layer to the pulsatile blood flow; and (2) in fewer cases, the rupture of the vasa vasorum leads to the weakening of the inner aortic wall [78]. In the first scenario, a false lumen derives from the progressive separation of the aortic wall layers, and its propagation leads to aortic rupture where the adventitia is disrupted: rupture quickly leads to exsanguination and death [79]. In the latter case, the bleeding results in intramural hematoma that may progress in aortic dissection. It has actually been hypothesized that a co-existence of these two conditions may, in turn, constitute a spectrum [80]. From a molecular point of view, dissection occurs as a consequence of the aortic-wall structure’s remodeling, due to inflammation and ECM-degradation processes. Once again, proteases exert an important role, since the infiltration of the activated macrophages and pro-inflammatory cytokines in the tunica media leads to an excessive production of MMP-1, MMP-9, and MMP-12 and to an imbalance between them and their inhibitors (TIMPs), which, in turn, results in the degradation of collagen and elastin fibers [81,82]. Wall remodeling is also maintained by VEGF-mediated neo-angiogenesis, as the production of VEGF (also functioning as a pro-inflammatory molecule) is increased in degraded medial layers [83].

Among classical risk factors associated with aortic dissection, namely older age, dyslipidemia, and increased levels of apolipoprotein A1, 80% of patients developing dissection have hypertension [84], which has a direct effect on the pathogenic mechanisms described above. Specifically, hypertension is demonstrated to promote a pro-inflammatory environment mainly by inducing macrophage recruitment and activation [85]; hypertensive patients, in fact, show high concentrations of VEGF, IL-6, MMP-2, and MMP-9 [86,87]. Other risk factors are recognized such as the male sex, a smoking habit, and the concurrence of connective tissue disorders such as MFS, LDS, vEDS, and BAV. Aortic dilatation is known to increase the risk of dissection, with the incidence complications reaching 30% at diameters > 60 mm [88]. Still, dilatation is proven not to be essential for developing a dissection, as ~60% of non-syndromic type A aortic dissection have diameters < 55 mm, while, in the absence of hypertension, MFS or BAV patients show a tendency to dissect at larger diameters [89,90]. Rare risk factors for aortic dissection are further represented by vascular inflammation due to autoimmune disorders such as Giant-cell arteritis, Takayasu Arteritis, Behçet disease, and systemic lupus erythematosus, while 1% to 5% of aortic dissections are secondary to aortitis [91].

### 3.2. Genetic Profiles of Dissection

To date, more than 30 TAAD-causative genes have been discovered (Table 1) and in the context of risk-prediction of TAA progression into potentially fatal dissection, it appears of primary interest to identify those genes or the specific type of variants/genetic profiles that, among others, are more prone to trigger and drive those processes eventually leading to sudden aortic-wall rupture. Marked in bold in Table 1, those genes were demonstrated to increase the risk of dissection at certain aortic sizes. Some of them represent causative genes of peculiar connective-tissue disorders described in association with TAA in its syndromic presentation. Pathogenic variants in FBN1, the MFS-causative gene (see Section 2.1), were found to increase the risk for Stanford type A and B dissection, even in the context of a normal or minimally dilated ascending aorta [92,93,94]. Indeed, a diagnosis of MFS is established in ~5% of patients with aortic dissection [95]. Haploinsufficiency, mainly resulting from truncating or splicing FBN1 mutations, is described as the leading mechanism behind the increased rate of aortic events [27]. Some overlapping cardiovascular clinical manifestations characterize MFS and LDS as basically reflecting a predisposition of patients affected by one or the other connective disorder to develop aneurysm and dissections of aorta and other arteries [92]. Implicated genes are *TGFBR1*, *TGFBR2*, and SMAD3, causing LDS type I and III, which have been associated with increased aortic risk of dissection at diameters < 50 mm [9]. *TGFBR1* and *TGFBR2* mutations’ carriers are often reported as a comparable clinical picture regarding presentation and natural history, even though clinical differences have actually been observed between the two populations of mutated subjects [96]. Regarding *TGFBR1* families, a gender-based difference in survival has been observed, resulting in significantly better outcomes in women than in men. Differences were also observed in terms of aortic diameter, with *TGBFR2* carriers dissecting at minimal aortic dilatation with respect to *TGFBR1* carriers in which the ascending aorta diameter at the time of type A dissections was 50 mm. A more recent and large multicenter retrospective registry of patients with genetically triggered thoracic aortic disease reported the data of 441 subjects harboring mutations on the TGF-β receptor genes, somewhat confirming the previous observations in *TGFBR2* mutations’ female carriers, that type A dissections of moderately dilated ascending aorta appeared more frequently than in males, which was not the case with *TGFBR1*, suggesting a more aggressive aortic disease in *TGFBR2* patients, especially in women [97]. Together with a *TGFBR2* mutation and the female sex, other features such as aortic tortuosity, hypertelorism, and translucent skin were found to be associated with an increased aortic dissection risk and may be taken into consideration in determining the optimal surgical timing (45 mm in the general population, lowered toward 40 in females with low body surface area, harboring a *TGFBR2* mutation, and presenting extra-aortic features). The literature data support the role of *SMAD3* as a dissection-predisposing gene [66,98,99,100], with mutations’ carriers having a cumulative risk of dissection or prophylactic surgical repair of 50% by age 50 and 85% by age 80. Interestingly, these subjects are characterized by a later onset of aortic events, possibly leading to a delayed diagnosis if compared, for instance, to MFS patients presenting with wider systemic features [101]. The early recognition of the disease, in this subset of patients, consequently lies in the family history of thoracic aortic disease (as a key element), so this observation remarks on the need to identify those subjects before dissection occurs. The majority (63%) of *SMAD3* mutations are missense and reside in the MH2 domain, which regulates the oligomerization with SMAD2 or SMAD4 and the subsequent activation of transcription, with those variants being associated with earlier aortic events compared to truncating, non-sense, gene-disrupting ones [102]. Even if generally associated with a specific connective-tissue disorder, it must be reasserted that *FBN1*, *TGFBR1*, *TGFBR2*, *SMAD3*, and *TGFB2* mutations also account for an additional 14% of non-syndromic familial TAA [40], in which the diagnosis of aortic disease may be complicated by the absence of peculiar systemic clinical features.

A vEDS-causing gene, COL3A1, is also among those referenced as dissection associated ones, due to the supporting literature data on population studies and case reports. The dissection was found to develop in different locations of the arterial tree, such as the abdominal aorta, as well as the iliac, coronary, and cervical arteries [103,104,105,106]. Concerning syndromic TAA in patients diagnosed as vEDS, few post-mortem cases were reported in 2010 [107], and 33 unrelated individuals or families were found to carry COL3A1 splicing mutations or small deletions partially removing splice-junctions sequences [108], and patients developing post-surgical or sudden aortic events are reported [109,110,111]. COL3A1 variants were additionally found to be associated with sporadic forms of TAAD in recent WES and case-control studies [112,113]. The type of variant involving the COL3A1 gene was also been suggested to correlate with the phenotype severity of vEDS; specifically, a subgroup of patients in a large European cohort bearing non-glycine missense and/or genetic variations at the C- and N-termini of type III procollagens was found to develop a later-onset and a milder phenotype with higher rates of aortic complications [114], while mutations at splice-donor sites were associated with higher mortality rates with respect to those involving the splice-acceptor sequences [115]. The literature data on animal models/human cohorts have identified a number of other genes, with mutations that were suggested to increase the risk of aortic dissection, such as EFEMP2 at the level of the ascending aorta [21], MYH11, ACTA2, and MAT2A in the thoracic aorta [43,116], and SLC2A10 at the aorta, as well as the arteries [44] LOX and PRKG1, with more limited data [28,117], and FOXE3 and MFAP5 as identified through WES studies [30,31] (see also Section 5).

## 4. Recommended Laboratory Workup for TAA/D Diagnosis and Risk Prediction

### 4.1. Medical History and Physical Examination

The 2014 ESC guidelines [11] stress the need to report any reference to a family history in the medical record in the initial evaluation along with the physical examination [118]. Silaschi and co-workers characterized some features that might be considered during the medical history’s collection as “red flags”, raising suspicion of acute aortic dissection, specifically: (a) known MFS or other connective tissue diseases; (b) family history; (c) known aortic valve disease; (d) known aortic aneurysm; and (e) previous aortic manipulation/surgery [76]. However, given that TAA patients are asymptomatic in the majority of cases, those few who present with premonitory symptoms have chest pain that should be ascribable to the presence of TAA where no other causes are found [76,119]. In the case of an aortic dissection occurring, however, the chest pain is characterized by a sudden onset and a more severe nature (sharp or “knife-like” pain, tearing, localized between the shoulder blades) often, but not invariably, manifesting after a strong physical or emotional strain [76,119]. The risk-assessment tool proposed by the 2010 North American guidelines is based on clinical data extracted from three groups of information, which are designated as the presence of (1) a “high-risk condition” (as MFS, family history of aortic disease); (2) “high-risk pain features” (severe chest pain); and (3) “high-risk examination features” (pulse deficit, aortic diastolic murmur). The resulting scoring system considers the mere number of those groups that are involved, ranging from 0 to 3, with the score being associated to an increased/decreased pre-test probability that influences, in turn, the diagnostic approach. Still, validation of this scoring system is still needed [120]. The diagnostic flow chart then combines the pre-test probabilities according to clinical data and the laboratory and imaging tests, as should be done in the clinical practice in emergency or chest-pain units [11].

### 4.2. “Traditional” Circulating Biomarkers for TAA/D

Biochemical markers currently do not play a major role in the overall diagnostic TAA flowchart and their support to diagnostic screening and decision-making, especially concerning time of intervention, is still limited. However, decades of studies have led to the identification and validation of a number of molecules that can assist physicians in: (a) identifying high-risk patients among those presenting with thoracic aortic aneurysm; (b) establishing prognostic stratification of individuals; and (c) evaluating aortic disease during the follow-up [121]. In addition, regarding the diagnostic workup described in the previous section, it has to be noticed that 5–13% of patients are classified at low risk for dissection and an even greater number of those who are classified at intermediate risk eventually turn out not to develop the complication [122]. Consequently, circulating biomarkers represent, in all respects, an appealing tool in assisting physicians, especially during the diagnostic algorithm proposed for aortic dissection. Among the potentially useful biomarkers, a number of studies have focused on D-Dimer (DD) evaluation, which became widely used in the clinical workup of suspected deep-vein thrombosis and pulmonary embolism due to its negative predictive value [123]. Its predictive power in identifying TAA patients at risk for developing dissection has been tested by several studies [124,125,126,127], showing DD to represent a highly sensitive yet largely unspecific biomarker, since DD levels increase in cancer infections, trauma, and surgery apart from deep-vein thrombosis, pulmonary embolism, and disseminated intravascular coagulopathy. However, negative DD could safely rule out aortic dissection, when associated with a sufficiently low pre-test probability of the disease [128]. DD might be used in prognosis prediction and in monitoring adverse events in patients in which the diagnosis of aortic dissection has been established [121].

Altered levels of metalloproteases and their inhibitors have been extensively studied in TAA samples (see Section 3.1), and their expression was also found to be different depending on the TAA etiology (atherosclerotic or non-atherosclerotic), size, growth rate, and location of the aneurysm (ascending or descending portion of the aorta) [129,130]. Concerning the potential role of these molecules as outcome predictors, preliminary data are available for MMP isoforms 1, 2, 3, 8, 9, 12, and 13. Among these, a low cutoff for plasma MMP8 has been correlated with ideal sensitivity and a negative predictive value for aortic dissection, suggesting a potential role in ruling out the occurrence of the disease. In addition, a combined use of MMP8 and DD has been proposed in ruling out aortic dissection in the diagnostic workup: as a matter of fact, the application of low cutoffs for both biomarkers was proven to be highly sensitive, and it allowed safe dissection’s being ruled out in 20% of patients [131]. A 2018 case-control study reported a close association between serum MMP9 and the existence of aortic aneurysms, suggesting a role for this isoform as a valuable marker for the discrimination of aortic aneurysm, especially for TAA [132]. Although suggestive, these data and those derived from MMP12, with levels that were found to be significantly increased in patients with Stanford type A aortic dissection with respect to coronary artery disease and control groups [133], are not yet sufficient to implement MMPs in clinical practice, so additional insights potentially coming from gene-expression studies are warranted [134].

Elastolytic process is augmented in TAA in which the elastin’s structure is modified, leading to increased levels of products of elastin degradation (soluble elastin fragments, sELAFs) and serum propeptide of type III procollagen, which were suggested as promising biomarkers for TAA [135]. Those were also found in patients developing aortic dissection, but their potential role as biomarkers is currently precluded due to the poorly measured sensitivity and specificity [136].

Increased C-reactive protein (CRP) levels have been observed in TAA, with respect to coronary artery disease patients and healthy controls [137], which may reflect the extensive inflammatory reaction and severe coagulopathies in individuals with acute type A aortic dissection and thoracic aortic aneurysm. In addition, CRP levels positively correlate with DD levels in aortic dissection [138], suggesting the presence of a strict relationship between these two factors in the pathophysiology of the complication and with plasma MMP8 and MMP9 in patients suspected of dissection [131]. These observations confirm the idea of a model in which inflammation, thrombosis, and ECM remodeling act synergically in driving the aortic rupture. However, the use of CRP does not apply in clinical practice as a specific diagnostic biomarker for aortic dissection, since increased levels can be observed in a number of other conditions (e.g., acute abdominal disease, pleuritis, pericarditis). A role for CRP may instead be considered for follow-up, as the stratification of patients with aortic dissection and studies have described an association between this acute-phase protein and the period of hospitalization [139]. CRP has been reported as an independent risk factor for in-hospital mortality [138]. Recently, Erdolu and as suggested the use of CRP and neutrophil to lymphocyte ratio values to predict mortality in patients with aortic dissections [140].

Sbarouni and co-workers analyzed 31 consecutive patients presenting acute aortic dissection showing higher homocysteine (Hcy) and lower folate compared to both chronic aneurysms and controls [141]. These data are not consistent with those resulting from studies on animal models with thoracic aortic dilatation, dissection, and rupture, in which hyperhomocysteinemia in the physiologic range did not induce/accelerate abnormal aortic growth in wild-type mice nor adverse pathologic progression in mice with an underlying predisposition for aortic dilatation [142]. An association has been found between elevated total Hcy (tHcy) levels and development of aortic dissection in a cohort of MFS patients [143] carrying the C677T polymorphism at the homozygous state. Although suggestive, especially for the marked remodeling of the extracellular matrix of the arterial wall induced by elevated Hcy through the activation of metalloproteases, further studies are needed to investigate the actual role of Hcy levels as a predictor of aneurysm progression [144].

Evidence was reported once again by a number of studies on MFS patients, underlying the increased levels of TGF-β as promoters of a significant weakening of the aortic wall resulting in TAA [145]. The usefulness of measuring TGF-β levels in such a specific population of patients as those affected by MFS, characterized by high variability in clinical manifestations, age of onset, and rate of aortic involvement, has been argued since the discovery of the alteration of this pathway in driving aortic complications observed in the disease [146]. This is of particular interest considering the potential advantages the evaluation of this plasma biomarker would represent in predicting disease severity and progression of artic dilatation. At the moment, the transition of this biomarker into the routine clinical evaluation of MFS and risk-stratification is not considered to be applicable, due to the inconsistency of results of different studies in retrieving elevated TGF-β levels in MFS patients; this is possibly due to the different population size, type of FBN1 mutation underlying the clinical picture and impacting the TGF-β pathway impairment, disease severity shown by patients, and use of medication [147]. Nonetheless, TGF-β concentration correlates with the level of aortic growth, faster aortic growth rate, previous aortic surgery, and acute aortic events during follow-up [148,149,150], suggesting its role as useful clinical marker in humans [151]. Acute elevations of TGF-β1 were found to be potentially predictive of aortic dissection in non-MFS patients [152], while the development of the complication has been associated with a significantly enhanced function of TGF-β1/Smad-signaling transduction, as a result of aortic remodeling incorporating both vascular injury and repair [153]; further evidence is emerging in this respect [154,155,156], suggesting an increased/unbalanced in plasma TGF-β in promoting aortic disease in different populations affected by genetic syndromes sharing aortic dilation as clinical complication, but this still has inconsistent results [157]. For those reasons, evaluation of this cytokine in the clinical practice of TAAD is not included in the 2014 ESC guidelines, unlike the molecular screening of those genes implicated in the TGF-β signaling.

Plasma levels of SMC protein have been evaluated in the context of aortic dissection. Among these, smooth muscle myosin heavy chain (smMHC) was first reported to acutely peak after aortic dissection [158] and later to be elevated in dissected patients compared with myocardial infarction subjects within 12 h from presentation or 3 h after onset [159,160].

Isozyme MM of creatine kinase (CK-MM, in which the protein is composed of two type M subunits) was observed to be selectively increased in aortic dissection patients 6 h after onset [161], while isozyme BB (2 type B subunits) was reported to peak at 12 h, in dissected patients with respect to controls, and return to normal within 24–36 h [162].

Calponin, a protein regulating myosin-actin interaction and SMCs contractility, was raised in serum of dissected patients in a study that enrolled multiple clinical centers [163], but its role in the diagnosis of dissection was eventually disregarded as the best results were obtained in early presenters and in Stanford Type A individuals.

More recently, smMHC, sELAF, D-dimer, or Polycystin 1 (PC1) alone were suggested as biomarkers for early diagnosis of acute aortic dissection, but the combination of these markers has been pointed out as having a significantly higher diagnostic value [164]. The application of these biomarkers in the current diagnostic flowchart has, however, not been considered.

Platelet activation and coagulopathy are also associated with aortic dissection and dissection extent [165]. A higher mean platelet volume/platelet (MPV/PLT) ratio was observed in aortic dissection patients along with a lower platelet count, which was also associated with an increased risk of in-hospital death before and after intervention; MPV/PLT, negatively associated with survival and platelet distribution, was established as a negative independent predictor of mortality in cohorts of dissected individuals [166,167]. Taken together, these data support the hypothesis of suppression of the PLT activation as future targets of therapy in acute aortic dissection, with particular reference to the prevention of systemic inflammation [165].

Data continue to emerge on potential biomarkers, e.g., aggrecan plasma levels were proposed as reliable biomarker to detect the presence of an acute type A aortic dissection in a very sensitive manner [168]; high-sensitivity cardiac troponin T concentration was proposed as an early biomarker for the risk stratification of patients with the same disease in the emergency department [169]; angiopoietin-like protein 8 (ANGPTL8), a hormone involved in the regulation of lipid metabolism and inflammation, combined with D-dimer and CRP was proposed as useful clinical predictor of TAAD [170]; and so on. Although promising, none of the abovementioned biomarkers showed a satisfactory profile for initial patient screening on large populations and case-control studies unavoidably provide limited information and need to be sustained by prospective enrolment of patients, such that further investigations are still necessary for implementing the guidelines, and novel, specific, and more readable biomarkers need to be unraveled [121]. To date, in fact, specific and promptly assessable biomarkers in the emergency departments worldwide are not available: the only validated biomarker is represented by DD, whose only validated cutoff for acute aortic syndromes is a 500 ng/mL fibrinogen equivalent unit (FEU) [121], the ESC guidelines indicating its measurement to rule out aortic dissection [11]. Still, as already mentioned, even in this case, the plasma biomarker lacks definitive specificity, as it may increase in several diseases including pericarditis, sepsis, and pulmonary embolism, and it may result falsely negative in conditions such as small intramural aortic hematomas or in those patients showing very early or very late symptoms of aortic dissection [121]. In this context, the broad-spectrum omic profiling (genomics, transcriptomics, metabolomics) may in the near future provide the necessary support (see Section 5) (Table 2).

### 4.3. Genetic Testing in Supporting TAA/D Diagnostics and in Risk Prediction: Where Do We Stand?

Although primarily considered as surgical disease, TAA’s optimal management greatly relies on an appropriate workup with the major purpose of identifying those features suggestive of a rapid progression of the aortic anomaly, thus predicting potentially life-threatening consequences of the disease. In this context, an accurate genetic evaluation/diagnosis serves different purposes: (a) guidance for overall medical management and surgical options; (b) timely evaluation of other organs that could be affected essentially in syndromic forms of TAA; (c) better definition of the prognosis; (d) identification of high-risk first-degree family members; (e) estimation of recurrence risk for future pregnancies in the prenatal diagnosis’ framework; and (f) support for imaging techniques in capturing nonsyndromic TAA patients who may be missed while developing dissection or rupture before reaching the guidelines-defined aortic diameter thresholds for aortic intervention [171]. As previously mentioned, syndromic and non-syndromic heritable thoracic aortic disease are, in most cases, inherited in an autosomal dominant manner except for rare X-linked and recessive conditions [172]. The accurate clinical evaluation of at-risk relatives is critical in this context, and ordinary and reproductive pre- and post-test genetic counseling allow for the early identification of an undiagnosed aortic disease in the first case and provide awareness about the risk of transmission to the offspring in the latter. Mutations are described to have variable penetrance depending on the TAA presentation, from almost 100% in MFS and 90% in LDS, to 50% in FTAAD and BAV in the presence of ascending aortic aneurysm. In fact, in the case of FTAAD, the causal mutation is found in much fewer cases (<10%) than in MFS or LDS, this discrepancy also being evident at the phenotypic level, presenting with a different severity of clinical manifestations along with age of presentation or diagnosis. When features of a connective-tissue disorder are present, patients should undergo genetic counselling and testing where appropriate [10]. The current ESC guidelines recommend genetic screening in first-degree relatives of TAA or aortic dissection and a diagnosis of familial aortic disease. In absence of a genetic diagnosis, at-risk relatives should undergo examination every 5 years. Screening should cover the entire arterial tree (including cerebral arteries) in families with nonsyndromic familial aortic disease [173]. According to the North American guidelines and related Class I recommendations, in case of identification of a mutation in one of the following genes, FBN1, TGFBR1, TGFBR2, COL3A1, ACTA2, and MYH11, which are associated with aortic aneurysm and/or dissection, first-degree relatives should undergo counseling and testing. Then, only the relatives with the genetic mutation should undergo aortic imaging. The guidelines provide some more recommendations (Class IIa and IIb): (a) ACTA2 sequencing should be considered in case of family history of thoracic aortic aneurysm and/or dissection; (b) TGFBR1, TGFBR2, and MYH11 sequencing may be considered in patients with a family history and clinical features associated with mutations in these genes; and (c) if one or more first-degree relatives of a patient with known thoracic aortic aneurysm and/or dissection are found to have thoracic aortic dilatation, aneurysm, or dissection, then referral to a geneticist may be considered [174]. Following the exclusion of a syndromic condition, nonsyndromic TAA, in which mutations in genes known to be involved in syndromic forms of TAAD are rarely found, may present suggestive features of a genetic etiology, which might include young age at presentation (<50 years old), multiple aneurysms or dissections, and aortic root aneurysm [175,176]. In this scenario, genetic counseling should begin with the collection of the most detailed information of a three-generation family history, for the presence of aneurysm, dissection, sudden deaths, and syndromic features that would help in determining the inheritance pattern, identifying at-risk relatives, and recognizing syndromic signs [172]. In 2009, Ripperger and co-workers reported three cases of sudden, unexpected death due to thoracic aortic dissection, pointing out the great benefit that could be derived from alerting the at-risk relatives of the deceased about a potential heritable etiology of the disease [177]. The authors propose the development of a standard procedure which includes genetic counseling for at-risk relatives and storage of DNA or unfixed tissue for molecular investigations that would eventually allow differential diagnostic reappraisal from a genetic point of view. In any case, during genetic consultation, patients should become aware of the limitations, benefits, and personal and familial implications of genetic testing. Besides, awareness should be raised on the possibility of a negative genetic test that would not necessarily exclude a genetic etiology, thus indicating the imaging to be performed anyways in the first-degree family members in the search for aortic disease [174]. In fact, some types of genetic variants may be undetectable by standard assays and, similarly, the causative mutation may involve a gene that has not yet been associated to TAAD, due to absence of data supporting the actual pathological effect of that variant [174]. As a matter of fact, regarding the most appropriate genetic test selection, no specific indications are provided by the European guidelines. Genetic-testing panels vary significantly among laboratories and despite the enthusiasm for the so-called “exome-first” approach in diagnosing such a complex disease as TAA, its actual benefit and routine application in the diagnostic workup currently represent a matter of debate within the international scientific community.

## 5. Latest Findings on TAA/D Genetic and Non-Genetic Biomarkers

### 5.1. RNA Signatures: A Novel, Noninvasive, and Promising Screening Option?

As discussed in the previous section, the identification of noninvasive approaches, which could support and extend the diagnostic/risk-prediction capabilities workup for TAAD, has been the subject of numerous studies that reported a number of molecules potentially driving the aneurysm formation and/or dissection. Yet, their real value lies in preclinical verification (in terms of sensitivity and specificity) and validation on large cohorts of patients vs. controls and in comparing subsets of patients that are affected by the disease but in which the presentation is highly variable. Recently, evidence is accumulating about the role of micro-RNAs (miRNAs), non-coding RNAs (ncRNAs), and circular RNAs in the development of many cardiovascular diseases, including aortic dissection [170]. Together, these molecules contribute to determining RNA-expression patterns and, ultimately, in defining the so-called “RNA signatures”, with the potential ability to accurately differentiate between different pathologic phenotypes (including the variable clinical manifestations of the same disease, as in the case of TAA), which has drawn the attention of numerous research groups during the last decades. This was also facilitated by the advancements in microarray and high-throughput technologies allowing the interrogation of different RNA populations in a single assay [178] (Table 3).

A 2007 study analyzed whole-genome gene-expression profiles from 94 peripheral blood samples (58 TAA subjects and 36 controls), identifying, with high accuracy (80% overall) a signature set of 41 biomarker genes specific of asymptomatic TAA patients [179]. This signature RNA test had also the ability to differentiate between ascending vs. descending aneurysms and between familial and sporadic TAA, also highlighting potential targets for intervention. Suggestions are derived from studies on miRNA profiles. The miR-29 family comprises three members, miR-29a/-29b/-29c, with expression that has been tested in a number of studies involving animal models and human normal/aneurysmatic/dissected aorta tissues [178]. Among these, miRNA-29b was found to play pivotal role in the formation of aneurysms [180,181], post-transcriptionally regulating the expression levels of multiple targets with a function in the ECM collagens, elastin, and fibrillin and modulating the aortic SMCs’ synthetic phenotype switch [182]. A therapeutic potential has been consequently proposed for miRNA-29b, with inhibition that could prevent TAA expansion. A cross-talk between miRNA-29b and TGF-β was observed, thus confirming its potential role in TAA, aortic dissection, and other diseases in which the disruption of the TGF-β signaling represents an underlying pathophysiological mechanism [178]. Two more miRNAs have been proposed as therapeutic targets for TAA, specifically miRNA-143 and miRNA-145, which appear to have a role in VSMCs’ phenotype switch [183] and, when up-regulated, an increased expression of VSMCs’ differentiation markers was observed [184].

**Table 3:** Novel potential circulating biomarkers for TAAD.

Marker	Animal Models	Human Cohort	TAA	TAAD
miR-1	-	aortic tissue specimens from ascending TAA patients (30)/3 tissues of patients with AAA, 11 tissues of patients with TAA and 8 controls [185,186]	+	
miR-21			+	
miR-29a			+	+
miR-133a			+	+
miR-15a	-	10 patients with TAA/3 tissue specimens from AAA patients, 11 from TAA patients and 8 controls/aortic tissue specimens from AAA patients (10) [186,187,188]	+	+
miR-22			+	+
miR-25			+	
miR-29b			+	
miR-125a-3p			+	
miR-126-3p			+	
miR-128			+	
miR-133b			+	+
miR-138-1			+	+
miR-142–5p			+	
miR-145			+	+
miR-146b-5p			+	
miR-183			+	+
miR-422a			+	
miR-433			+	+
miR-486–5p			+	
miR-487b			+	
miR-491–3p			+	+
miR-553			+	+
miR-638			+	
miR-940			+	+
miR-193a-3p			+	+
miR-768–5p			+	+
miR-886–5p			+	+
miR-195			+	+
miR-140–5p			+	+
miR-30e			+	+
miR-101			+	+
miR-744			+	+
miR-193a-5p			+	+
miR-30c	-	3 tissues specimens from AAA patients, 11 from TAA patients and 8 controls [186]	+	
miR-155			+	
miR-204			+	
miR-143	mouse models [183]	-	+	+

AAA: abdominal aortic aneurysm; miR: microRNA; TAA: aortic aneurysm, thoracic.

Both miRNAs were down-regulated in aorta from acute aortic dissection patients [189], while their expression levels appeared increased or decreased in TAA depending on the study [183,188]. Other mi-RNAs were suggested to modulate aortic SMC towards the differentiation process (miR-1 [190], miR-133 [191], miR-663 [192], miR-424 [193], miR-195 [194], miR-138 [192]), de-differentiation process (miR-221/-222 [195], miR26a [196], miR-146a [197], miR-155 [198], miR-31 [199], miR-181b [200]), and phenotype switch mechanisms (miR-21 [201], miR-24 [202]) (Table 3). The involvement of all these molecules in aortic SMCs biology and ECM integrity maintenance and composition, as well as their differential expression in aneurysmatic tissues with respect to controls, supports their potential role in TAAD pathogenesis. Still, miRNAs actively promoting those processes leading to aortic dilatation and from aortic dilatation to aortic dissection need to be clearly differentiated from simple bystanders, in order to identify precise therapeutic targets [178]. A validation study was carried out by Moushi and co-workers to verify the literature data on the association between a large number of miRNAs using plasma from TAA patients collected before and after surgery [203]. In this study, where 24 papers and 11 miRNAs were selected for validation, miR-193a-5p and miR-30-b-5p were found to be down-regulated in plasma samples collected before the aneurysm removal with respect to post-surgical ones, making these molecules the most promising among those tested. A recent study analyzed 19 TAA patients and 19 controls allowing the identification of 232 differentially expressed miRNAs, among which miR-574-5p was proposed as a potential therapeutic target [178]. A cohort of 40 MFS patients undergoing elective ascending aorta surgery were enrolled in a study conducted by D’Amico and co-workers, which aimed to compare TAA histomorphological features, a miRNA profile and related target genes, and to find specific alterations that may explain the earlier and more severe clinical outcomes in MFS patients [204]. Twenty-five miRNAs, including miR-26a, miR-29, miR-143, and miR-145, were found to be downregulated, while miR-632 was upregulated in MFS/TAA in vivo; in addition, 28 upregulated and 7 downregulated genes were identified, some of them belonging to the CDH1/APC and CCNA2/TP53 signaling pathways, which the authors propose to be further tested as potential therapeutic targets to counteract the rapid progression of MFS aortopathy.

Studies have also addressed a potential role of long ncRNAs (lncRNAs) in modulating TAA development and progression acting via several molecular pathways. With regards to known pathophysiological mechanisms underlying TAA onset, including aortic-wall expansion and VSMCs’ dedifferentiation from the contractile to the synthetic phenotype, which is regulated by lncRNAs’ CARMN, LUCAT1, SMILR, and MALAT1, Patamsytė and co-workers tested these molecules in clinical aortic tissue and blood-plasma samples from TAA and non-TAA patients using the qRT-PCR method [205], attributing to LUCAT1 alone the ability to discriminate aneurysmal disease in patients’ blood plasma and a diagnostic potential for TAA. Subsequently, high-throughput sequencing was used to analyze lncRNAs’ expression profile in human thoracic aortic dissection, revealing a set of dysregulated lncRNAs and predicting their multiple potential functions in the disease (lnc_1421, ENSG00000269936, lncRNA XIST, NSG00000248508, ENSG00000226530, EG00000259719 [206]). Further evidence continues to emerge, as LncRNA Sox2ot was shown to modulate TAA progression by regulating miR-330-5p/Myh11, which was suggested as new potential mode to treat TAA [207], as well as lncRNA CDKN2B-AS1, which was found to aggravate the pathogenesis of human thoracic aortic dissection [208], or LncRNA Xist’s contributing to arterial smooth muscle cell apoptosis through the miR-29b-3p/Eln pathway [209]. Additional data have been reported on lncRNA H19, which has the ability to regulate smooth muscle cell functions participating in the development of aortic dissection through sponging miR-193b-3p [210], and on lncRNA OIP5-AS1, which exacerbates aorta intima, media, and adventitia injury in the development of aortic dissection, through the upregulation of TUB via sponging miR-143-3p [211].

Circular RNAs (CircRNAs) represent endogenous lncRNAs with regulatory roles at both the transcriptional and post-transcriptional levels, by binding and interacting with miRNAs. Among them, circRNA-101238 was found to be highly expressed in human thoracic aortic dissection specimens, leading to a lower expression of the downstream target miR-320a, and, in turn, to increased MMP9 expression [212], while circMARK3-miR-1273-Fgr interaction was suggested to have a certain clinical significance in human acute Stanford type A aortic dissection (AAAD) tested by RNA-seq [213].

Although promising, miRNA research needs to be extended to other cellular components besides VSMCs and endothelial cells, such as macrophages and fibroblasts; other ncRNAs, such as repeat-associated small interfering RNAs (rasiRNAs) and Piwi-interacting RNAs (piRNAs), may also be involved in the disease and should undergo further investigation; strategies for local and safe ncRNA or miRNA delivery in patients are required as well as the development of in vivo imaging techniques mimicking those effects that were observed in animal models [214]. In this respect, it seems that there is still a long way to go and a lot to be done in order to test these hypotheses and to include RNA markers in the routinely diagnostic flowchart for TAA.

### 5.2. Novel Genes and the WES Outbreak: Pros and Cons in the Clinical Practice and Applicability

As is well known, the high-throughput molecular technologies’ outbreak in the mid-2000s, represented an actual “revolution” in the genetic research and diagnostic fields for Mendelian disorders as well as those pathologies recognizing, among other factors, a genetic etiology. Still, the opportunity to interrogate the entire codifying component of the human genome (WES) or the entire human genome itself (Whole Genome Sequencing (WGS)) in a single assay, at a decreasing cost and in a multiplex mode, even if unarguably beneficial for gene-discovery purposes, led to a debate over its actual usefulness and applicability in the routinely diagnostic flowchart for a series of pathologies in the place of targeted gene/gene panels approaches. Regarding TAA, disease-specific gene panels are expanding in terms of the number of included genes. That is especially due to the phenotypic heterogeneity and allelic overlap between syndromic and non-syndromic cases [31]. Positive genetic testing for TAA has important implications for disease management and for at-risk family members screening, so that guidance in decision-making about when to re-test, as panels continue to expand, is necessary.

Back in 2014, WES analysis identified a new mutation in TGFB2 involved in a familial case of non-syndromic aortic disease [215]. As discussed in Section 3.1, TGFB2, together with FBN1, TGFBR1, TGFBR2, and SMAD3 mutations, accounts for 14% of non-syndromic familial TAA [40], but current American Guidelines do not include this gene among those that should be tested in patients with a TAA family history [174]. WES identified recurrent gain-of-function mutations in PRKG1 as causative for thoracic aortic aneurysms and acute aortic dissections [117], meanwhile MAT2A, LOX, and FOXE3 have been suggested as predisposing genes [28,30,43]. Milewicz and co-workers applied WES to identify causative mutations in novel genes for TAAD [216]. Their strategy resided in sequencing distant relatives with TAAD, in order to reduce the large number of rare variants identified using WES, and filtering for heterozygous rare variants that are shared between relatives and that are predicted to disrupt protein function and segregate with the TAAD phenotype in other family members. This strategy led to the successful identification of novel genes for FTAAD, which were validated through minimal additional molecular, cellular, or animal studies. Despite the advantages of this approach in gene discovery, the authors also stressed the importance of the molecular, cellular, and animal studies required to link the gene variant to the disease phenotype that also needs the development of novel techniques and the establishment of new collaborations, with this last issue to be especially encouraged. WES was also applied in an attempt to identify the causative mutation in two young patients with acute type B aortic dissection without syndromic features, who were afterwards diagnosed with MFS as they were found to carry two pathogenic FBN1 mutations [217]. In this work, the authors emphasized the necessity of genetic testing for young patients with type B aortic dissection through WES, which is a timely, robust, and inexpensive technique for genetic sequencing, particularly for TAAD that is caused by numerous genes, in order to identify syndromic conditions, such as MFS, which has a diagnosis that may facilitate periodic surveillance, prophylactic surgical measures, and genetic counseling. Another study performed WES on 183 FTAAD families without significant systemic features of MFS identifying FBN1 mutations in 11 families, suggesting the screening of this gene in this category of FTAAD subjects [218]. WES was used as routine genetic test in 102 TAAD patients [219], allowing for the opportunity for the personalized management of patients tailored to the specifics of the genetic mutation (e.g., prophylactic surgical interventions earlier and at smaller aortic for malignant ones). Candidate genetic modifiers of syndromic and familial TAA severity were identified through WES in a cohort of 27 subjects with syndromic or familial TAA and presenting extreme phenotypes (ADCK4 and COL15A1 genes [220]). Authors speculate that these hypotheses-generating findings initiate a path toward risk stratification through genetic testing at an early stage of disease and identify novel therapeutic targets, pinpointing WES as the most suitable technology for those purposes. However, they also point out the need to develop novel statistical and experimental platforms to define how specific variants interact to actually influence phenotype. New genes (MLX, DAB2IP, EP300, ZFYVE9, PML, PRKCD) were suggested as candidate aortic dissection-associated genes, through correlation analyses performed on the results of a WES study on a cohort of 99 Chinese cases [112]. Variants of TES and other focal adhesion scaffold genes were shown to predispose individuals to isolated TAA, by affecting vascular smooth-muscle-cell phenotypic modulation and vasoconstrictive function [221]. These results were obtained from a WES study on a cohort of 551 sporadic isolated TAA cases and 1071 controls, which expanded the genetic landscape across this disease, showing focal adhesion scaffold genes as a novel category of TAA causal genes. The application of different methodologies (other than WES, such as Next Generation Sequencing (NGS) panels and mouse models), led to the identification of a number of other suggestive genes for TAA/D (Table 4).

A flowchart for a dedicated screening program for relatives of patients affected by nonsyndromic TAA was proposed by Faggion Vinholo and co-workers, in which genetic screening is strongly supported by the literature data [6]. In all these studies, authors list the advantages of applying WES in a TAA clinical setting: (1) it is comprehensive, including (but not limited to) testing all the 30-plus TAAD genes that have been identified to date; (2) the WES of the proband permits straightforward, comprehensive screening of the family members by less complex and less-costly single-site (Sanger) sequencing and non-mutation-carrying family members can be spared repeated imaging studies; and (3) patients with no mutation of a known TAAD gene, especially those with affected family members, are likely to provide a clinical “gold mine” for mining WES data for totally novel TAAD genes, especially in light of the genes identified to date that only account for approximately 30% of aortic diseases in nonsyndromic TAAD patients [219].

On the other hand, Renard and co-workers reported the results of a study aimed to accurately identify TAAD-predisposing genes using the Clinical Genome Resource (ClinGen) framework [36]. Fifty-three candidate genes were analyzed, nine of which were categorized as “TAAD genes with definitive or strong gene disease-association” (ACTA2, COL3A1, FBN1, MYH11, MYLK, SMAD3, TGFB2, TGFBR1, TGFBR2), while eight were defined as “potentially diagnostic genes, with moderate or limited gene-disease association”, as they could allow diagnosis of thoracic aortic enlargement but are primarily associated with other clinical features and do not carry a significant risk for dissection (EFEMP2, ELN, FBN2, FLNA, NOTCH1, SLC2A10, SMAD4, SKI). The other genes had limited or no clinical evidence for TAA/D. These findings led the authors to question the usefulness of very large panels (and, as a consequence, WES) that instead increase the risk for ambiguous and even false-positive reports and raise the probability of identifying variants of uncertain significance (VUS), which may cause unnecessary distress for patients and family members and create diagnostic confusion, misallocation of resources for follow-up, and even unjustified genetic discrimination [228]. A gene panel containing the most established genes associated with the highest risk of early fatal complications in patients with hereditary TAAD (ACTA1, COL3A1, FBN1, MYH11, SMAD3, TGFB2, TGFBR1, TGFBR2, MYLK) was evaluated as sufficient to identify the prevailing majority of variants most likely to be causative of the disease, with respect to the larger panel including 174 genes [37]. The authors suggest that a curated list of genes associated with heritable TAAD has the ability to identify patients and families at risk and reduce inconclusive diagnostic testing by limiting the scope of screened genes. The quality of a diagnostic panel should be judged on the clinical validity of genes included in that panel rather than on the number. In this respect, efforts need to be made in the direction of more complete and uniform genetic profiles with greater power to diagnose TAAD and predict its fatal consequences [36]. However, data analysis of a WES experiment could definitely focus on the most suspected genes and/or on those with a role in causing/modulating TAA and its complications, which is supported by the most robust data, and then broaden the evaluation in case of negative results. The probability of incidental findings and VUSs remains high and this issue should probably be addressed in determining the most appropriate way of discussing it during genetic counseling.

## 6. Conclusions

TAA’s bad reputation of “silent killer” is to be ascribed to its characteristic features, including its slow and gradual formation and the absence of visible signs, with patients remaining asymptomatic. This condition is elusive and yet potentially life-threatening, as it manifests itself only once the aneurysm is large enough to lead to an acute and devastating aortic event, with a significant percentage of patients dying before reaching the hospital. As a result, it is of the utmost relevance to identify biomarkers for the early identification of asymptomatic patients, a task which is both essential and challenging. In this regard, there’s an important distinction to be made between the TAD management within the emergency department, in which the room to maneuver is objectively limited, and those other situations in which the fatal event has not happened yet. In the first case, as Mehta and co-workers pointed out in a very recent review, the margin of intervention is essentially directed to improving the patient’s outcome by different means, including the multidisciplinary collaboration between specialists (emergency physicians, surgeons, radiologists) and identification of the optimal interventional treatment and post-operative care [229]. These goals are basically pursued by an accurate evaluation of the aortic pathology, the “nature of the pain” described by patients, the malperfusion at different locations (neurologic, spinal cord, visceral, renal), and the correct interpretation of diagnostic-imaging findings, while laboratory tests are essentially represented by basic metabolic panels, cardiac biomarkers, DD, troponin T, and complete blood count; those measurements, however, are insufficient in acute aortic pathologies diagnosis and, in most cases, are non-specific. On the other hand, for risk-stratification, risk-prediction in first-degree family members, disease management, and follow-up purposes, the identification of affordable and reliable markers is critical, challenging, and yet practicable. In this respect, efforts have been directed over the years to the evaluation of circulating biomarkers that could be used in the TAA diagnostic path to discriminate those patients who are more prone to develop a potentially fatal complication. A number of circulating biomarkers have been suggested to be useful, alone or in combination, essentially in ruling out aortic dissection (DD, MMP8, smMHC, sELAF, PC1). According to the 2014 European guidelines, in case of the suspicion of aortic dissection, part of these molecules is currently tested, yet they have not entered the clinical arena [11]. Traditional circulating biomarkers do not represent a satisfactory and reliable support in the initial patient screening as well, in which, on the contrary, the molecular/genetic evaluation can be diriment. Genetic testing, especially that which interrogates several genes at once in a parallel approach that is, at present, undoubtedly preferable to the cascade one, has long been included in the diagnostic flowchart for TAA diagnosis. First of all, it allows for the identification of a co-existing condition with TAA, such as MFS or LDS, thus directing the most appropriate management in terms of periodic check-ups, time of intervention, risk-recurrence calculation for pregnancies, and screening for first-degree relatives. In addition, the constant implementation of molecular methodologies allowing for the interrogation of the entire genome or transcriptome in an “omic” approach could be, undoubtedly, beneficial for patients’ stratification. In fact, the combination of the data derived from the WES/WGS/RNA-seq approaches can help define profiles that could be highly specific for subgroups of TAA patients, not to mention the potential use of those data in deepening knowledge about the disease’s onset and progression as well as for identifying new targets for therapy. Even with the considerable limitations characterizing the omic approaches (production of a large amount of bioinformatic data that need to be correctly interpreted, safely stored, and validated through functional studies; the possibility of VUSs and incidental findings), the future benefits they may represent for the improvement of the TAA diagnostic work-up have to be considered and perhaps should be addressed more closely and in greater detail in the international guidelines.

## Figures and Tables

**Table 1:** Genes associated with TAA/D (syndromic and non-syndromic).

Biological Process/Cellular Compartment	Gene	Protein	OMIM	Syndromic TAA/D	Non-Syndromic FTAA/D	Associated Syndrome/Diseases
**Extracellular matrix/remodeling**	***BGN***	Biglycan	300,989	+	−	Meester-Loeys syndrome. ARD, TAAD, pulmonary artery aneurysm, IA, arterial tortuosity [19].
	***COL3A1***	Collagen Type III α1 Chain	130,050	+	−	EDS, vascular type IV. TAAD, early aortic dissection, visceral arterial dissection, vessel fragility [20].
	*EFEMP2*	EGF Containing Fibulin Extracellular Matrix Protein 2	614,437	+	−	Cutis laxa, AR type Ib. Ascending aortic aneurysms, other arterial aneurysms, arterial tortuosity, stenosis [21].
	*ELN*	Elastin	123,700 185,500	+	−	Cutis laxa. AD ARD, ascending aortic aneurysm and dissection [22], TAA [23,24], BAV, IA possibly associated with SVAS.
	***FBN1***	Fibrillin-1	154,700	+	+	Marfan syndrome. ARD, TAA [25], TAAD [26], AAA, other arterial aneurysms, pulmonary artery dilatation, arterial tortuosity [27].
	***LOX***	Protein-lysine 6-oxidase	617,168	−	+	AAT10. AAA, hepatic artery aneurysm, BAV, CAD, TAAD [28,29].
	*MFAP5*	Microfibril Associated Protein 5	616,166	−	+	AAT9. ARD, TAA [30,31].
**Smooth muscle cells**	***ACTA2***	Smooth muscle α-actin	611,788 613,834 614,042	+	+	AAT6, multisystemic smooth muscle dysfunction, MYMY5. Early aortic dissection, CAD, stroke (moyamoya disease), PDA, pulmonary artery dilation, BAV, TAAD, TAA [24,32].
	*FLNA*	Filamin A	300,049	+	−	Periventricular nodular heterotopia and otopalatodigital syndrome. Aortic dilatation/aneurysms, peripheral arterial dilatation, PDA, IA, BAV, TAA [32,33].
	***MYH11***	Smooth muscle myosin heavy chain	132,900	−	+	AAT4. PDA, CAD, peripheral vascular occlusive disease, carotid IA, TAAD, early aortic dissection [32,34,35].
	***MYLK***	Myosin light chain kinase	613,780	−	+	AAT7. TAAD, early aortic dissections [36,37].
**TGF-β signaling**	***LTBP1***	Latent TGF-β binding protein 1	150,390	+	−	Aortic dilation with associated musculoskeletal findings. Dental anomalies, short stature. TAAD, AAA, visceral and peripheral arterial aneurysm [38].
	***LTBP3***	Latent TGF-β binding protein 3	602,090			
	*SMAD2*	SMAD2	619,657 619,656	+	-	Unidentified CTD with arterial aneurysm/dissections. ARD, ascending aortic aneurysms, vertebral/carotid aneurysms and dissections [39], AAA.
	***SMAD3***	SMAD3	613,795	+	+	LDS type III. ARD, TAAD [40], early aortic dissection [39], AAA, arterial tortuosity, other arterial aneurysms/dissections [9], IA, BAV.
	***SMAD4***	SMAD4	175,050	+	-	JP/HHT syndrome. ARD, TAAD [39], AVMs, IA.
	*SMAD6*	SMAD6	602,931	-	+	AOVD2. BAV/TAA [24].
	***TGFB2***	TGF-β2	614,816	+	+	LDS type IV. ARD, TAA [40], TAAD, arterial tortuosity [39], other arterial aneurysms, BAV.
	***TGFB3***	TGF-β3	615,582	+	-	LDS type V. ARD, TAAD, AAA/dissection, other arterial aneurysms, IA/dissection [39].
	***TGFBR1***	TGF-β receptor type 1	609,192	+	+	LDS type I+AAT5. TAAD [40], early aortic dissection, AAA, arterial tortuosity, other arterial aneurysms/dissection [9], IA, PDA, BAV.
	***TGFBR2***	TGF-β receptor type 2	610,168	+	+	LDS type II+AAT3. TAAD [40], early aortic dissection, AAA, arterial tortuosity, other arterial aneurysms/dissection [9], IA, PDA, BAV.
**Others**	*AXIN1/PDIA2 locus*	−	−	+	−	BAV. BAV/TAA [41].
	*FBN2*	Fibrillin-2	121,050	+	−	Contractual arachnodactyly. Rare ARD and aortic dissection [42], BAV, PDA.
	***FOXE3***	Forkhead box 3	617,349	−	+	AAT11. TAAD [30] (primarily type A dissection).
	*MAT2A*	Methionine adenosyl-transferase II α	n.a.	−	+	FTAA Thoracic aortic aneurysms [30,43]. BAV.
	***NOTCH1***	NOTCH1	109,730	−	+	AOVD1. BAV/TAAD [24].
	***PRKG1***	Type 1 cGMP-dependent protein kinase	615,436	−	+	AAT8. TAAD [28,43], early aortic dissection, AAA, coronary artery aneurysm/dissection, aortic tortuosity, small vessel, CVD.
	*ROBO4*	Roundabout guidance receptor 4	607,528	−	+	BAV. BAV/TAA [24].
	*SKI*	Sloan Kettering proto-oncoprotein	182,212	+	−	Shprintzen–Goldberg syndrome. ARD, arterial tortuosity, pulmonary artery dilation, other (splenic) arterial aneurysms [36].
	*SLC2A10*	Glucose transporter 10	208,050	+	−	Arterial tortuosity syndrome. ARD, ascending aortic aneurysms [36], other arterial aneurysms, arterial tortuosity [44], elongated arteries, aortic/pulmonary artery stenosis.

**In bold**: genes associated with dissection. AAA: abdominal aortic aneurysm; AAT/TAA: aortic aneurysm, thoracic; AD: autosomal dominant; AOVD: aortic valve disease; ARD: aortic root dilatation; AVM: arteriovenous malformation; BAV: bicuspid aortic valve; CAD: coronary artery disease; CTD: connective tissue disease; CVD: cerebrovascular disease; EDS: Ehlers-Danlos syndrome; FTAA: familial thoracic aortic aneurysm; FTAAD: familial thoracic aortic aneurysm and/or dissection; HHT: hereditary hemorrhagic telangiectasia; IA: intracranial aneurysm; JP: juvenile polyposis; LDS:, Loeys-Dietz syndrome; n.a.: not applicable; PDA: patent ductus arteriosus; SVAS: supravalvular aortic stenosis; TGF: transforming growth factor; TAAD: thoracic aortic aneurysm and/or dissection.

**Table 2:** Proposed/suggested circulating biomarkers for TAAD.

Marker	Animal Models	Human Cohort	TAA	TAAD
ANGPTL8	-	78 patients with AD and 72 controls [170]		
Calponin	-	217 patients with AD [163]	+	+
CK-BB	-	10 patients with AAD [162]		
CK-MM	-	22 patients with AAD [161]		
CRP	-	49 patients with aortic disorders [130]	+	+
	-	114 patients with AAD [139]		
	-	118 patients with AAD [140]		
CSPCP (aggrecan)	-	33 patients with AAD [168]	+	+
cTnT	-	103 patients with AAD [169]		
DD	-	24 patients with AD/TAAD [124]		+
	-	64 patients with AD [125]		
	-	220 patients with AAD [126]		
Hcy	-	31 patients with AAD [141]		+
	C57BL/6J mice [142]	-		
MMP8	-	186 patients suspected AAD [131]		
MMP9	-	105 patients with AAA, 79 with TAA, 112 controls [132]	+	
MMP12	-	15 patients with AAD, 10 controls [133]		
MPV/PLT	-	300 patients with aortic disorders [166]		+
	-	183 patients with AAD [167]		
sELAFs	-	62 patients with AAA [135]		
	-	25 patients with AAD [136]		
smMHC	Mice [158]			
	-	27 patients with AD [159]		+
TIMP1	-	93 patients with TAA and 24 controls [70]	+	
TIMP2	-	93 patients with TAA and 24 controls [70]	+	
TGF-β	-	50 families with LDS [145]		+
	-	28 patients with AAD [152]		
	-	40 patients with aortic disorders [153]	+	+
	-	1 patient with LDS [155]	+	

AAA: abdominal aortic aneurysm; AAD: acute aortic dissection; AD: aortic dissection; ANGPTL8: angiopoietin-like protein 8; CK-BB: isozyme BB of creatine kinase; CK-MM: isozyme MM of creatine kinase; CRP: C-reactive protein; CSPCS: cartilage-specific proteoglycan core protein; cTnT: cardiac troponin T; Hcy: homocysteine; LDS: Loeys–Dietz syndrome; MMP: metalloproteinase; sELAFs: soluble elastin fragments; smMHC: smooth muscle myosin heavy chain; TAA: aortic aneurysm, thoracic; TAAD: thoracic aortic aneurysm and/or dissection.

**Table 4:** Novel potential TAAD-associated genes.

Study/Methodology	Genes Identified	Animal Models	Human Cohort
**WES (WHOLE EXOME** **SEQUENCING)**	*MLX*, *DAB2IP*, *EP300*, *ZFYVE9*, *PML*, *PRKCD*	-	99 patients with TAA [112]
	*ADCK4*, *COL15A1*	-	27 patients with fTAA [220]
	*TES*, *TLN1*, *ZYX*	C57/BL6 mice	556 patients with sporadic TAA and 1092 controls [221]
	*MCTP2*	-	151 patients with TAAD [222]
	*16p13.1* duplication	-	1 patient with fTAAD [223]
	*C1R*	-	13 patients with BAV [224]
**NGS PANELS**	*SCARF2*	-	810 cases of suspected TAA [225]
**MOUSE MODELS**	*ADAM17*	Sm22α-Cre mice [226]	-
	*RBBP8*	Male C57/BL6 mice	12 Aortic aneurysm/dissection samples [227]

AAT/TAA: aortic aneurysm, thoracic; BAV: bicuspid aortic valve; FTAAD: familial thoracic aortic aneurysm and/or dissection; TAAD: thoracic aortic aneurysm and/or dissection.

## Data Availability

Not applicable.

## Abbreviations

AAA	abdominal aortic aneurysm
AAAD	acute Stanford type A aortic dissection
ADAMTS	A Disintegrin and Metalloproteinase with Thrombospondin motifs
BAV	bicuspid aortic valve
circRNAs	circular RNAs
CK-MM	Creatine-kinase isozyme MM
CRP	C-reactive protein
DD	d-Dimer
ECM	extracellular matrix
ESC	European Society of Cardiology
FTAAD	familial thoracic aortic aneurysm and dissection
Hcy	homocysteine
LAP	latency-associated peptide
LDS	Loeys-Dietz syndrome
lncRNAs	long non-coding RNAs
LTBP	latent TGF? binding protein
MFS	Marfan syndrome
miRNAs	micro-RNAs
MMPs	metalloproteinases
MPV	mean platelet volume
ncRNAs	non-coding RNAs
NGS	next-generation sequencing
PC1	Polycistin 1
piRNAs	Piwi-interacting RNAs
PLT	platelet
qRT-PCR	Quantitative Reverse Transcription Polymerase Chain Reaction
rasiRNAs	repeat associated small interfering RNAs
sELAFs	soluble elastin fragments
SMCs	smooth muscle cells
smMHC	smooth muscle myosin heavy chain
TAA/D	thoracic aortic aneurysm and dissection
TAA	thoracic aortic aneurys
TAD	thoracic aortic dissection
TEVAR	thoracic endovascular aortic repair
TGFβ	Transforming Growth Factor-β
tHcy	total homocysteine
TIMPs	tissue inhibitors of metalloproteinases
vEDS	vascular Ehlers-Danlos syndrome
VEGF	vascular endothelial growth factor
VSMCs	vascular smooth muscle cells
VUS	variant of uncertain significance
WES	whole exome sequencing
WGS	whole genome sequencing
